# Multidomain interventions: state-of-the-art and future directions for protocols to implement precision dementia risk reduction. A user manual for Brain Health Services—part 4 of 6

**DOI:** 10.1186/s13195-021-00875-8

**Published:** 2021-10-11

**Authors:** Alina Solomon, Ruth Stephen, Daniele Altomare, Emmanuel Carrera, Giovanni B. Frisoni, Jenni Kulmala, José Luis Molinuevo, Peter Nilsson, Tiia Ngandu, Federica Ribaldi, Bruno Vellas, Philip Scheltens, Miia Kivipelto, on behalf of the European Task Force for Brain Health Services, Marc Abramowicz, Daniele Altomare, Frederik Barkhof, Marcelo Berthier, Melanie Bieler, Kaj Blennow, Carol Brayne, Andrea Brioschi, Emmanuel Carrera, Gael Chételat, Chantal Csajka, Jean-François Demonet, Alessandra Dodich, Bruno Dubois, Giovanni B. Frisoni, Valentina Garibotto, Jean Georges, Samia Hurst, Frank Jessen, Miia Kivipelto, David Llewellyn, Laura Mcwhirter, Richard Milne, Carolina Minguillón, Carlo Miniussi, José Luis Molinuevo, Peter M. Nilsson, Janice Ranson, Federica Ribaldi, Craig Ritchie, Philip Scheltens, Alina Solomon, Cornelia van Duijn, Wiesje van der Flier, Bruno Vellas, Leonie Visser

**Affiliations:** 1 Institute of Clinical Medicine, University of Eastern Finland, Kuopio, Finland; 2 Division of Clinical Geriatrics, NVS, Karolinska Institutet, Stockholm, Sweden; 3 Ageing Epidemiology Research Unit, School of Public Health, Imperial College London, London, UK; 4 Laboratory of Neuroimaging of Aging (LANVIE), University of Geneva, Geneva, Switzerland; 5 Memory Clinic, Geneva University Hospitals, Geneva, Switzerland; 6 Stroke Center, Department of Neurology, University Hospitals and University of Geneva, Geneva, Switzerland; 7 Department of Public Health Solutions, Public Health Promotion Unit, Finnish Institute for Health and Welfare, Helsinki, Finland; 8 Faculty of Social Sciences, Tampere University, Tampere, Finland; 9 Barcelonaβeta Brain Research Center (BBRC), Pasqual Maragall Foundation, Barcelona, Spain; 10 Department of Clinical Sciences, Skåne University Hospital, Lund University, Malmö, Sweden; 11 Laboratory of Alzheimer’s Neuroimaging and Epidemiology (LANE), Saint John of God Clinical Research Centre, Brescia, Italy; 12 Department of Molecular and Translational Medicine, University of Brescia, Brescia, Italy; 13 Gérontopole of Toulouse, University Hospital of Toulouse (CHU-Toulouse), Toulouse, France; 14 Alzheimer Center Amsterdam, Department of Neurology, Amsterdam Neuroscience, Vrije Universiteit Amsterdam, Amsterdam UMC, Amsterdam, The Netherlands; 15 Institute of Public Health and Clinical Nutrition, University of Eastern Finland, Kuopio, Finland

**Keywords:** Brain Health Services, Dementia, Aging, Alzheimer’s disease, Prevention, Dementia risk, Risk reduction

## Abstract

Although prevention of dementia and late-life cognitive decline is a major public health priority, there are currently no generally established prevention strategies or operational models for implementing such strategies into practice. This article is a narrative review of available evidence from multidomain dementia prevention trials targeting several risk factors and disease mechanisms simultaneously, in individuals without dementia at baseline. Based on the findings, we formulate recommendations for implementing precision risk reduction strategies into new services called Brain Health Services. A literature search was conducted using medical databases (MEDLINE via PubMed and SCOPUS) to select relevant studies: non-pharmacological multidomain interventions (i.e., combining two or more intervention domains), target population including individuals without dementia, and primary outcomes including cognitive/functional performance changes and/or incident cognitive impairment or dementia. Further literature searches covered the following topics: sub-group analyses assessing potential modifiers for the intervention effect on cognition in the multidomain prevention trials, dementia risk scores used as surrogate outcomes in multidomain prevention trials, dementia risk scores in relation to brain pathology markers, and cardiovascular risk scores in relation to dementia. Multidomain intervention studies conducted so far appear to have mixed results and substantial variability in target populations, format and intensity of interventions, choice of control conditions, and outcome measures. Most trials were conducted in high-income countries. The differences in design between the larger, longer-term trials that met vs. did not meet their primary outcomes suggest that multidomain intervention effectiveness may be dependent on a precision prevention approach, i.e., successfully identifying the at-risk groups who are most likely to benefit. One such successful trial has already developed an operational model for implementing the intervention into practice. Evidence on the efficacy of risk reduction interventions is promising, but not yet conclusive. More long-term multidomain randomized controlled trials are needed to fill the current evidence gaps, especially concerning low- and middle-income countries and integration of dementia prevention with existing cerebrovascular prevention programs. A precision risk reduction approach may be most effective for dementia prevention. Such an approach could be implemented in Brain Health Services.

## Background

Although prevention of dementia and late-life cognitive decline is a major public health priority, there are currently no generally established prevention strategies or operational models for implementing such strategies into practice [1]. During the past 20 years, epidemiological studies have pointed out several modifiable risk factors for dementia, including cardiovascular, metabolic, and lifestyle-related factors (e.g., hypertension, hyperlipidemia, diabetes, obesity, physical inactivity, unhealthy dietary habits, smoking, excessive alcohol consumption, social isolation) [2]. In 2019, the World Health Organization (WHO) published the first guidelines for risk reduction of cognitive decline and dementia [3]. The guidelines were developed to provide evidence-based recommendations on interventions aiming to delay or prevent the onset of cognitive decline and dementia. The reviewed evidence covered interventions including physical activity, tobacco cessation, nutrition, cognitive training, social activity, interventions for alcohol use disorders, and management of weight, hypertension, diabetes, dyslipidemia, depression, and hearing loss [3].

According to the WHO, these risk reduction guidelines are targeted primarily at healthcare providers working at a first- or second-level facility or at the district level, including basic outpatient and inpatient services. While the WHO has pointed out several key considerations for implementation, it is not yet fully clear exactly how the recommendations should be tailored to specific populations, as well as different healthcare system contexts. Due to the complex multifactorial etiology of dementia, and variations in risk factors between different individuals and populations, a “one-size-fits-all” approach to prevention is not going to work. The current risk reduction guidelines are also based on interventions targeting single risk factors. However, overall dementia risk is most often the result of a combination of risk and protective factors that may have different contributions in different individuals or at different life stages. Thus, a precision risk reduction approach is most likely to be effective, i.e., tailoring the right interventions for the right people and at the right time. Operational models for the risk reduction interventions would also have to take into account the local or national specifics of both public health policies and healthcare systems.

Early identification of at-risk individuals is an essential part of the precision risk reduction approach. Many multifactorial dementia risk scores have already been developed for the early identification of at-risk individuals who may also benefit most from preventive interventions [4]. Although such risk scores could in principle facilitate precision risk reduction by, e.g., highlighting an individual’s specific combination of risk factors and facilitating more tailored interventions, the majority of such risk scores are not yet sufficiently validated and/or have not been tested in actual prevention trials. In addition, dementia shares many risk factors with other chronic diseases such as cardiovascular conditions (CVD), diabetes, or stroke. Validated risk scores for such conditions are already used as part of the established prevention programs [5]. However, it is not clear to what extent vascular/diabetes risk scores could be useful in the context of dementia prevention and facilitate the integration of dementia prevention within other established prevention programs.

This article is a narrative review of available evidence from multidomain dementia prevention trials targeting several risk factors and disease mechanisms simultaneously, in individuals without dementia at baseline. A key aspect of the evidence review concerns the use of dementia and CVD risk scores in such prevention trials. Based on the findings, we formulate some practical recommendations for implementing precision risk reduction strategies (see Table 1) into new services called Brain Health Services (BHSs). Currently, dementia prevention falls under the domain of memory clinics. However, the current memory clinics have been designed for the needs of patients with overt cognitive and/or behavioral disorders and are ill-equipped to deal with a population of cognitive unimpaired individuals and their growing demand for dementia prevention and cognitive enhancement interventions [6]. We envision the development of new BHSs, with specific missions including dementia risk profiling [7], dementia risk communication [8], dementia risk reduction (the present paper), and cognitive enhancement [9] and with specific societal challenges [10]. This will be the fourth part of a Special Issue series of six articles, published in *Alzheimer’s Research & Therapy*, which together provide a user manual for BHSs.

**Table 1:** Recommendations for practical implementation of precision dementia risk reduction interventions

**1. Target populations** • A risk reduction intervention should not be applied unselectively (focus on various at-risk groups). • At-risk groups should be preferably selected using validated risk scores or algorithms. • The most suitable risk score or algorithm should be carefully chosen to fit the purpose, e.g., stage of the risk/disease continuum, age group, level of cognitive performance, and type of intervention to be applied. For example, for multidomain lifestyle interventions, the risk score/algorithm should select individuals with the type of risk profile that the intervention aims to modify. • Risk reduction interventions should preferably start early, before substantial brain pathology and cognitive/functional impairment have already occurred. • People with genetic susceptibility for dementia (e.g., based on *APOE* ε4 genotype) may also benefit from early risk reduction interventions. This should be further investigated in intervention studies. **2. Interventions** • Multidomain interventions (targeting several risk factors and disease mechanisms simultaneously) may be needed for an optimal dementia risk reduction. • Interventions should (i) do the right things and (ii) do enough for them, i.e., target an individual’s overall risk profile with sufficient intensity to produce an effect. Only general healthy lifestyle advice may not be enough, and a more structured intervention program should be proposed. • Intervention content should be adapted to local/national risk context (e.g., some risk factors may be more prevalent/severe in some countries than others) and various settings and integrated with other chronic non-communicable diseases risk reduction programs when feasible. • Radical lifestyle changes may be difficult to both initiate and maintain longer term. Smaller changes gradually introduced across multiple lifestyle domains may facilitate long-term adherence. • As the social component is important, group sessions and/or group activities should be facilitated when feasible. • New technology may facilitate effective, personalized, and feasible interventions and implementation (eHealth and mHealth). • Intervention effects should be monitored. Risk scores could be useful for this purpose as well, if they include modifiable factors and are sufficiently sensitive to change over time.	

## Multidomain interventions

### Effects of multidomain interventions on cognition and related outcomes

An English-language literature search was conducted using medical databases (MEDLINE via PubMed and SCOPUS, until December 2020) and keywords such as “multidomain,” “intervention,” “dementia,” “cognition,” “cognitive decline,” and “risk reduction.” The following criteria were used to select relevant studies: non-pharmacological multidomain interventions (defined as combining two or more intervention domains), target population including individuals without dementia at baseline, and primary outcomes including cognitive/functional performance and/or incident mild cognitive impairment (MCI) or dementia. The 14 identified studies are summarized in Table 2.

**Table 2:** Overview of multidomain intervention trials for the prevention of cognitive decline and dementia

Study	Design and population	Multidomain intervention	Primary outcome	Main results
The MAX trial (Barnes et al., 2013) [11]	*N* = 126 Adults with cognitive complaints, USA Age, 65+ years Duration, 12 weeks	Individual, home-based mental activity plus class-based physical activity—4 groups 1. Intervention (mental activity + exercise vs. 2. Intervention + control (mental activity intervention + exercise control) vs. 3. Control + intervention (mental activity control + exercise intervention) vs. 4. Control (mental activity + exercise)	Global cognitive change based on a comprehensive neuropsychological test battery	Physical plus mental activity was associated with significant improvements in global cognitive function.
Alves et al., 2013 [12]	*N* = 56 Healthy women, Brazil Mean age, 66.8 years Duration, 24 weeks	Creatine supplementation and exercise—4 groups 1. Creatine supplementation vs. 2. Placebo vs. 3. Creatine supplementation + strength training vs. 4. Placebo + strength training	Cognitive function (memory, selective attention, and inhibitory control)	No significant effect on cognition.
Ihle-Hansen et al., 2014 [13]	*N* = 195 Patients after the first stroke, Norway Mean age, 71.6 years Duration, 12 months	Outpatient stroke nurse and physician consultation 3 and 6 months post-stroke, information about lifestyle and brain health. Medical treatment optimized. Tailored advice regarding risk factor management and treatment plan sent to a general practitioner. Offered smoking cessation courses vs. care as usual	Trail-making test A and 10-word test from baseline to 12 months follow-up	No difference between the intervention and control groups.
The SMART study (Fiatarone Singh et al., 2014) [14]	*N* = 100 Adults with MCI, Australia Mean age, 70.1 years Duration, 18 months	2 supervised interventions, 2–3 days/week for 6 months with 18 months follow-up - Active OR sham physical training (high-intensity progressive resistance training vs. seated calisthenics) plus - Active OR sham cognitive training (computerized, multidomain cognitive training vs. watching videos/quizzes)	Global cognitive function (ADAS-Cog) and functional independence	Resistance training significantly improved global cognitive function, with the maintenance of executive and global benefits over 18 months.
Lam et al., 2015 [15]	*N* = 555 Adults with MCI, Hong Kong Mean age, 75.4 years Duration, 18 months	Physical exercise vs. Cognitive activity vs. Integrated cognitive and physical exercise vs. Social activity (active control) groups	Clinical Dementia Rating sum of boxes (CDR-SOB) scores	No difference between the groups for change in CDR-SOB and functional scores. Integrated physical and cognitive intervention exerted significantly better cognitive benefits on category verbal fluency test but not across all cognitive domains compared to single cognitive or physical activity intervention.
FINGER (Ngandu et al., 2015) [16]	*N* = 1260 Persons at-risk of dementia, Finland Age, 60 to 77 years Duration, 2 years	Lifestyle intervention (diet, exercise, cognitive training, vascular risk monitoring) vs. general health advice	Cognition on the neuropsychological test battery	Significant intervention benefit on cognition.
ASPIS (Matz et al., 2015) [17]	*N* = 202 Stroke patients, Austria Age, 40 to 80 years Duration, 2 years	Multidomain intervention (clinical therapy, adequate blood pressure, lipid and glycaemic control, healthy diet, regular physical activity, cognitive training) vs. standard stroke care	Cognition on Alzheimer Disease Assessment Scale and neuropsychological test battery	No difference between the intervention and control groups.
preDIVA (Moll van Charante et al., 2016) [18]	*N* = 3526 Community-dwelling older persons, the Netherlands Age, 70 to 78 years Duration, 6 years	Multidomain intensive vascular care vs. standard care	Incident dementia and disability score	No difference between the intervention and control groups.
MAPT (Andrieu et al., 2017) [19]	*N* = 1680 Community-dwelling older persons, France Mean age, 75.3 years Duration, 3 years	1. Multidomain intervention + omega-3 supplementation 2. Multidomain intervention + placebo 3. Omega-3 supplementation alone 4. Placebo alone	Cognitive decline on composite *Z* score	No difference between the intervention and control groups.
Look AHEAD (Espeland et al., 2018) [20]	*N* = 1091 Overweight or obese adults with type 2 diabetes, USA Age, 45 to 76 years Duration, 10 years	Lifestyle intervention (diet modification and physical activity) yielding long-term weight loss vs. support and education	Change in cognition (composite measure)	No difference between the intervention and control groups.
KENKOJISEICH (Bae et al., 2019) [21]	*N* = 83 Individuals with MCI, Japan Mean age, 76 years Duration, 24 weeks	Physical, cognitive, and social activity sessions vs. health education	Cognition on the National Center for Geriatrics and Gerontology Functional Assessment Tool	Significant intervention effect on spatial working memory.
Blumenthal et al., 2019 [22]	*N* = 160 Older adults with cognitive impairment and no dementia, USA Mean age, > 55 years Duration, 6 months	Diet and exercise—4 groups: 1. Aerobic exercise vs. 2. DASH diet nutritional counseling vs. 3. Combination of both aerobic exercise and DASH vs. 4. Health education	Global measure of executive cognitive functioning	The largest improvements were observed for combined aerobic exercise and DASH diet group.
Body Brain Life for Cognitive Decline (McMaster et al., 2020) [23]	*N* = 119 Subjective cognitive decline or mild cognitive impairment, Australia Age, 70 to 78 years Duration, 8 weeks	Educational modules covering dementia and lifestyle risk factors, Mediterranean diet, physical activity, and cognitive engagement and additional active components: dietitian sessions, an exercise physiologist session, and online brain training vs. 4 online informational modules to reduce dementia risk	Dementia risk using the Australian National University-Alzheimer’s Disease Risk Index (ANU-ADRI) and cognition	The intervention group showed a significantly lower ANU-ADRI score and a significantly higher cognition score than the control group.
DO-HEALTH (Bischoff-Ferrari et al., 2020) [24]	*N* = 2157 Adults having no major health events in the 5 years prior to enrolment, sufficient mobility, and good cognitive status, Europe (Switzerland, France, Germany, Portugal, and Austria) Age, 70 years or older Duration, 3 years	Supplementation and exercise—8 groups: 1. 2000 IU/day of vitamin D3, 1 g/day of omega-3s, and a strength-training exercise program vs. 2. Vitamin D3 and omega-3s vs. 3. Vitamin D3 and exercise vs. 4. Vitamin D3 alone vs. 5. Omega-3s and exercise vs. 6. Omega-3s alone vs. 7. Exercise alone vs. 8. Placebo	6 primary outcomes: change in systolic and diastolic blood pressure, Short Physical Performance Battery (SPPB), Montreal Cognitive Assessment (MoCA), and incidence rates of non-vertebral fractures and infections	No statistically significant benefits of any intervention individually or in combination for all 6 end points.

*ADAS-Cog* Alzheimer’s Disease Assessment Scale-Cognitive Subscale, *ANU-ADRI* Australian National University Alzheimer’s Disease Risk Index, *ASPIS* Austrian Polyintervention Study to Prevent Cognitive Decline After Ischemic Stroke, *CDR-SOB* Clinical Dementia Rating sum of boxes, *DASH* Dietary Approaches to Stop Hypertension, *FINGER* Finnish Geriatric Intervention Study to Prevent Cognitive Impairment and Disability, *MAPT* Multidomain Alzheimer Preventive Trial, *MAX* Mental Activity and eXercise, *MoCA* Montreal Cognitive Assessment, *preDIVA* Prevention of Dementia by Intensive Vascular Care, *SMART* Study of Mental and Resistance Training, *SPPB* Short Physical Performance Battery

Most of the trials were conducted in high-income countries. There was a substantial variability in the target populations, format and intensity of the interventions, choice of control conditions, and outcome measures. Recruited participants were aged between 40 and 80 years and varied from relatively unselected primary care populations to general populations with risk factors for dementia, and patients with MCI. The sample size ranged from 56 to 3526 participants and duration of the intervention from 8 weeks to 10 years (1 year or longer in 9 out of 14 trials). The interventions included intensive lifestyle programs offering various combinations of diet advice, dietary supplements, physical exercise advice and/or training programs, cognitive training, and management of vascular/metabolic risk factors. The intervention groups were compared to standard care, placebo, general information/health advice, or sham exercises.

Overall, the results appear to be mixed. Smaller (*N* < 160 participants) and/or shorter trials (up to 24 weeks) seemed more likely to report intervention benefits on overall cognition and some specific domains (e.g., spatial working memory, executive functioning). Of the 5 larger (*N* > 1000 participants) and longer-term trials (at least 2 years), only the Finnish Geriatric Intervention Study to Prevent Cognitive Impairment and Disability (FINGER) reported significant intervention benefits on the primary and secondary cognitive outcomes [16]. The results from these 5 trials are difficult to compare directly due to substantial differences in, e.g., target populations, format and intensity of the interventions, and outcome measures. However, several characteristics specific for the FINGER intervention model have been emphasized as potential reasons behind its cognitive benefits [25]: (i) selection of an at-risk older population (60–77 years) based on the validated Cardiovascular Risk Factors, Aging and Dementia (CAIDE) Risk Score [26]; (ii) multidomain intervention covering five domains, i.e., diet, exercise, cognitive training, social activities, and monitoring of vascular/metabolic risk; and (iii) more intensive intervention, e.g., inclusion of an exercise program at the gym in addition to advice on physically active lifestyle and inclusion of both individual and group sessions to ensure sufficient support and motivation for healthy lifestyle changes.

### Risk stratification in multidomain intervention trials

A cursory look at the mixed findings shown in Table 2 may tempt clinicians into thinking that the multidomain intervention concept is not as promising as initially hypothesized. However, the differences in the design between larger, longer-term trials that met vs. did not meet their primary outcomes suggest that multidomain intervention effectiveness may be highly dependent on a precision prevention approach, i.e., successfully identifying the at-risk groups who are most likely to benefit. To further investigate this, another literature search was conducted focusing on sub-group analyses assessing the potential modifiers for the intervention effect on cognition in the multidomain prevention trials listed in Table 2. Identified sub-group analyses were based primarily on the FINGER, Multidomain Alzheimer Preventive Trial (MAPT), and Prevention of Dementia by Intensive Vascular Care (preDIVA) trials. Several of these analyses were pre-specified in the trial protocols, while others were conducted post hoc. The results are summarized in Table 3.

**Table 3:** Examples of sub-group analyses assessing the potential modifiers for the intervention effect on cognition in multidomain prevention trials

Multidomain trials	Study	Potential intervention effect modifiers	Analyses	Results
FINGER	Rosenberg et al., 2018 [27]	Sex, age, education, socioeconomic status, cognition, cardiovascular factors, and cardiovascular comorbidity at baseline	Pre-specified	No significant differences in cognitive intervention benefits by sex, age, education, socioeconomic status, cognition, cardiovascular factors, and cardiovascular comorbidity.
	Solomon et al., 2018 [28]	APOE ε4 allele	Pre-specified	Intervention benefits were not significantly different between carriers and non-carriers. Clear benefit in APOE4 carriers in stratified analyses.
	Deckers et al., 2020 [29]	LIBRA index at baseline	Post hoc	Participants with a higher LIBRA index at baseline had overall less cognitive improvement over time, but this effect was not different between the intervention and control groups.
	Sindi et al., 2017 [30]	Leukocyte telomere length	Post hoc	More pronounced cognitive intervention benefits in individuals with shorter baseline leukocyte telomere length (higher-risk individuals).
	Stephen et al., 2019 [31]	Brain volumes and cortical thickness	Post hoc	More pronounced cognitive intervention effects in individuals with higher brain baseline cortical thickness and volumes.
MAPT	Andrieu et al., 2017 [19]	Cognition and functioning level at baseline	Pre-specified	No significant differences in intervention effects.
	Andrieu et al., 2017 [19]	APOE ε4 allele	Post.hoc	Intervention effects were not significantly different between carriers and non-carriers.
	Tabue-Teguo et al., 2018 [32]	Frailty status	Post hoc	Beneficial effects of multidomain intervention and n3 PUFA supplementation on cognition did not differ between frail and non-frail participants.
	Delrieu et al., 2019 [33]	Amyloid status	Post hoc	Multidomain intervention alone or in combination with omega-3 fatty acids was associated with improved primary cognitive outcomes in individuals with positive amyloid status.
	Chhetri et al., 2018 [34]	CAIDE score ≥ 6 points	Post hoc	High-risk subjects for dementia screened with CAIDE dementia score might benefit more from multidomain intervention.
preDIVA	Moll van Charante et al., 2016 [18]	Participants free from cardiovascular disease	Pre-specified	Participants with a history free from cardiovascular disease who were adherent to the intervention had a significantly lower risk of dementia compared to the control group.
	Moll van Charante et al., 2016 [18]	Untreated hypertension at baseline	Pre-specified	Participants with untreated hypertension who were adherent to the intervention had a significantly lower risk of dementia compared with the control group.
	van Middelaar et al., 2018 [35]	LIBRA index at baseline	Post hoc	LIBRA modifiable dementia risk score did not identify a (high-)risk group in whom the multidomain intervention was effective in preventing dementia or cognitive decline.

Subgroup analysis type (pre-specified and post hoc) was assessed from published trial protocols

*APOE* apolipoprotein E, *CAIDE* Cardiovascular Risk Factors, Aging and Dementia, *FINGER* Finnish Geriatric Intervention Study to Prevent Cognitive Impairment and Disability, *LIBRA* LIfestyle for BRAin health, *MAPT* Multidomain Alzheimer Preventive Trial, *preDIVA* Prevention of Dementia by Intensive Vascular Care, *PUFA* polyunsaturated fatty acids

In the FINGER trial, where participants were selected using the CAIDE Dementia Risk Score including age, sex, education, hypertension, hypercholesterolemia, obesity, and physical inactivity, the intervention seemed to be beneficial for cognition irrespective of further stratification by sociodemographic, cognitive, or cardiovascular factors [27]. Although participants with a higher LIfestyle for BRAin health (LIBRA) index at baseline had overall less cognitive improvement over time, this effect was not different between the intervention and control groups [29]. The LIBRA index is based on 12 modifiable risk factors [36] that partly overlap with those included in the CAIDE score, which may explain this result.

Interestingly, significant benefits on cognition were reported among participants in the MAPT trial with a CAIDE score ≥ 6 points (the same cutoff used in FINGER) [34]. Other analyses stratified by frailty status found no differences in the intervention effect on cognition between frail and non-frail MAPT participants [32].

The LIBRA index did not identify high-risk individuals in whom the preDIVA intervention was beneficial [35]. However, preDIVA trial participants with untreated hypertension and who were adherent to the intervention had a significantly lower risk of dementia compared with the control group [18]. This is perhaps not surprising considering that the preDIVA intervention placed more weight on the cardiovascular risk management component compared with the lifestyle components. Participants without a history of cardiovascular disease who were adherent to the preDIVA intervention also had a significantly lower risk of dementia compared to the control group.

The impact of genetic factors on the intervention effects on cognition has been so far reported only in the FINGER and MAPT trials. No significant difference in the intervention-related cognitive benefits was observed between *APOE* ε4 allele carriers and non-carriers. However, analyses stratified by *APOE* ε4 carrier status showed a significant intervention-related cognitive benefit among the group of ε4 carriers in FINGER [28], with a similar trend in MAPT [19]. In addition, a more pronounced cognitive benefit was reported in FINGER participants with shorter leukocyte telomere length at baseline, i.e., higher-risk individuals [30]. However, it would be particularly important for multidomain prevention trials to assess the impact of genetic risk beyond APOE genotype alone, e.g., via polygenic risk scores.

Brain imaging markers were also considered as potential intervention effect modifiers in the FINGER and MAPT trials. The MAPT intervention was reported to be associated with beneficial effects on cognition in individuals with amyloid positivity on positron emission tomography (PET) scans [33]. However, the FINGER intervention had more cognitive benefits in participants with higher brain volumes and cortical thickness at baseline [31]. It has been suggested that, while amyloid PET detects the early stages of amyloid deposition, morphological changes on MRI generally occur later in the Alzheimer’s disease (AD) continuum [37]. In this context, the MAPT and FINGER findings emphasize that the best window of opportunity for precision risk reduction may be among individuals who have an increased dementia risk, but not yet substantial brain pathology and/or substantial cognitive/functional impairment. In other words, earlier and better targeted multidomain interventions may be most effective.

### Estimating dementia risk reduction in early multidomain interventions

The AD continuum is characterized by a long period (up to decades) between the start of brain pathology and dementia onset [38]. In early interventions targeting at-risk individuals without substantial impairment, and with clinical trial durations that only very rarely exceed 2–3 years, dementia is not a feasible trial outcome. In the absence of direct data on the impact of multidomain interventions on reduction in dementia incidence, other ways to estimate the risk reduction are needed. Multifactorial risk scores that provide standardized, evidence-based estimates for the risk of dementia may be particularly useful for this purpose and may also facilitate continuous monitoring of the intervention effects in practice by both clinicians and at-risk individuals.

Dementia risk scores have only recently started to be used in the context of prevention trials. For example, the FINGER trial used the CAIDE score for the recruitment of at-risk participants [16]. Several of the larger, longer-term multidomain intervention trials with cognition or dementia as primary outcomes are now also testing dementia risk scores as potential surrogate outcomes for estimating intervention effects on dementia risk reduction.

Table 4 summarizes the dementia risk scores used as outcome measures in multidomain prevention trials, including those where cognitive performance or dementia is not the primary outcome. Two smaller and shorter-term trials with younger individuals, Body Brain Life [23] and the In-MINDD feasibility trial [39], have used a dementia risk score as the primary outcome. In the larger and longer-term trials, dementia risk scores have been used as outcomes in post hoc analyses.

**Table 4:** Dementia risk scores used as surrogate outcomes in multidomain prevention trials

Study	Trial	Dementia risk score	Outcome type	Main results
O’Donnell et al., 2015 [39]	In-MINDD	LIBRA	Primary	Participants in both arms of the trial showed a small improvement in their LIBRA score. The improvement was slightly larger in the intervention arm, but not statistically significant after 6 months.
Solomon et al., 2018 [40]	FINGER	CAIDE	Post hoc	The intervention had a significant impact on lowering the CAIDE risk score after 2 years.
Barbera et al., 2020 [41]	FINGER MAPT preDIVA	CAIDE	Post hoc, individual participants pooled analysis	CAIDE score decreased significantly as a result of the interventions after 2 years.
Coley et al., 2020 [42]	preDIVA MAPT HATICE	LIBRA and CAIDE	Post hoc, each trial analyzed separately	CAIDE and LIBRA scores showed statistically significant between-group differences after multidomain interventions after 1.5 to 2 years.
Deckers et al., 2020 [29]	FINGER	LIBRA	Post hoc	The intervention decreased dementia risk as indicated by decreasing LIBRA score after 2 years.
McMaster et al., 2020 [23]	Body Brain Life	ANU-ADRI	Primary	Significant reduction in ANU-ADRI score for BBL compared with control after 2 months.

*ANU-ADRI* Australian National - University Alzheimer’s Disease Risk Index, *CAIDE* Cardiovascular Risk Factors, Aging and Dementia, *FINGER* Finnish Geriatric Intervention Study to Prevent Cognitive Impairment and Disability, *HATICE* Healthy Ageing Through Internet Counselling in the Elderly, *In-MINDD* Innovative Midlife Intervention for Dementia Deterrence, *LIBRA* LIfestyle for BRAin health, *MAPT* Multidomain Alzheimer Preventive Trial, *preDIVA* Prevention of Dementia by Intensive Vascular Care

Overall, the results indicate significant intervention benefits on the tested dementia risk scores, supporting the potential use of these scores for estimating dementia risk reduction. However, estimates from such analyses are currently difficult to interpret or compare between different risk scores and would have to be verified against direct data on dementia incidence following the intervention. A potential solution for this could be extended follow-ups of trial participants after the intervention is completed, e.g., via healthcare registries if not otherwise feasible.

### Dementia risk scores and brain pathology markers

Although many dementia risk scores have been developed for predicting subsequent dementia or cognitive decline, only two have so far been tested in relation to brain pathology (e.g., cerebrospinal fluid (CSF) or neuroimaging biomarkers, or brain pathology at autopsy). Detailed knowledge on the performance of a dementia risk score in predicting specific types of brain pathology (e.g., AD-related, or cerebrovascular) is essential for making informed decisions about the intervention study design, e.g., identification of the appropriate at-risk individuals who are most likely to benefit from a specific intervention, or monitoring of intervention effects on dementia risk reduction.

An English-language literature search was conducted using medical databases (MEDLINE via PubMed and SCOPUS, until December 2020) and keywords such as “dementia,” “Alzheimer,” “risk score,” “risk algorithm,” “biomarker,” “MRI,” “PET,” and “pathology.” The focus was on dementia risk scores including modifiable factors. A summary of the reported relations between dementia risk scores and brain pathology markers is shown in Table 5. The CAIDE score is so far the most extensively tested in relation to biomarkers, including CSF and neuroimaging markers (structural MRI and amyloid PET), and post-mortem brain pathology. The Australian National University Alzheimer’s Disease Risk Index (ANU-ADRI) score has been tested in relation to MRI markers.

**Table 5:** Dementia risk scores in relation to brain pathology markers

Risk score	Study design	Biomarkers	Findings
**CAIDE**			
Vuorinen et al., 2015 [43]	Cohort, general population, Finland *N* = 181 Mean age, 50 years Follow-up, 30 years	Brain cortical thickness, white matter lesions, medial temporal atrophy on MRI	Higher score associated with higher medial temporal atrophy, white matter lesions, and lower cortical thickness two to three decades later.
Enache et al., 2016 [44]	Cohort, memory clinic patients SCI and MCI, Sweden *N* = 724 Age, > 40 years Follow-up, cross-sectional	AD-related CSF markers	Higher score associated with CSF markers of neurodegeneration (↓Aβ and ↑total tau).
Stephen et al., 2017 [45]	Cohort, at-risk for dementia, Finland *N* = 132 Age, 60–77 years Follow-up, 20–30 years	Brain volumes and cortical thickness, medial temporal atrophy, white matter lesions on MRI, and amyloid positivity on PiB-PET	Higher score associated with lower volumes and cortical thickness, medial temporal atrophy, and white matter lesions but not with amyloid on PiB-PET.
Hooshmand et al., 2018 [46]	Cohort, without dementia at baseline, Finland *N* = 149 Age, ≥ 85 years Follow-up, 10 years	Brain pathology at autopsy	Higher score associated with increased cerebral infractions.
O’Brien et al., 2019 [47]	Cohort, middle-aged healthy adults, UK *N* = 149 Age, 40–59 years Follow-up, 2 years	Rate of change in brain and ventricular volumes on MRI	Higher score associated with progressive brain atrophy rates.
**ANU-ADRI**			
Cherbuin et al., 2019 [48]	Cohort, individuals free of dementia, Australia *N* = 461 Age, 60–64 years Follow-up, 12 years	Total and regional brain volumes on MRI	Higher score was associated with lower cortical gray matter particularly in the default mode network.

*Aβ* amyloid-beta, *AD* Alzheimer’s disease, *ANU-ADRI* Australian National University Alzheimer’s Disease Risk Index, *CSF* cerebrospinal fluid, *MRI* magnetic resonance imaging, *PiB-PET* Pittsburgh compound B-positron emission tomography, *MCI* mild cognitive impairment, *SCI* subjective cognitive impairment

Although neuropathology markers can be used directly as predictors of dementia risk, currently available markers (CSF and neuroimaging) are more difficult to assess outside highly specialized memory clinic settings, and their use is not always recommended in a population of cognitively unimpaired individuals for ethical or health economics reasons [49]. Validating simpler and easier to use dementia risk scores in relation to neuropathology markers would thus offer more cost-effective solutions for early identification of at-risk individuals in a broader range of clinical settings, where risk reduction interventions can also be started earlier, before the onset of substantial impairment requiring referral for more invasive and costly diagnostic procedures.

Another key aspect to consider when choosing a dementia risk score for precision risk reduction is to what extent it captures risk versus prevention potential, i.e., room for improvement with intervention. Risk scores such as CAIDE, ANU-ADRI, or LIBRA include modifiable risk factors, thus indicating not only the risk profile, but also the intervention components that are needed to modify an individual’s risk profile. It is currently unclear to what extent neuropathology markers could be used to estimate prevention potential, although they could be very useful as secondary outcomes in multidomain interventions that combine non-pharmacological approaches with disease-modifying drugs. Assessing the neuropathology markers in multidomain prevention trials could also provide valuable knowledge on the interplay between cognitive reserve and brain pathology in determining intervention outcomes.

## Dementia vs. cardiovascular risk reduction

The 2019 WHO guidelines for risk reduction of cognitive decline and dementia also covered evidence on interventions targeted at reducing cardiovascular risk factors (e.g., hypertension, dyslipidemia, and diabetes) both pharmacologically and non-pharmacologically. The potential for integrating these recommendations into existing cardiovascular prevention programs was also emphasized. Although validated CVD risk scores have long been an established part of cardiovascular prevention, the testing of CVD risk scores in the context of dementia prevention has only recently started.

For example, the Framingham CVD risk score includes age, sex, systolic blood pressure, treatment for hypertension, HDL cholesterol, total cholesterol, smoking, and diabetes. The Framingham stroke risk score combines age, systolic blood pressure, treatment for hypertension, diabetes, smoking, prior CVD (myocardial infarction, angina pectoris, coronary insufficiency, intermittent claudication, or congestive heart failure), atrial fibrillation, and left ventricular hypertrophy. Both versions of the Framingham risk score at midlife have been reported to predict cognitive decline and dementia [50]. Additionally, the Framingham CVD risk score has been reported to predict vascular dementia [51] and clinical progression in patients with AD dementia, particularly in those with genetic and atherosclerotic risk factors [52]. However, the Framingham CVD risk score was not associated with structural brain measures on MRI [53].

The Framingham CVD risk score and two dementia risk scores (CAIDE and Washington Heights-Inwood Columbia Aging Project (WHICAP)) were investigated in relation to cognitive performance in different ethnic groups [54]. All three scores were significantly associated with cognition in both Hispanic/Latino and non-Hispanic/Latino populations.

Life’s Simple 7 (LS7), defined by the American Heart Association as the 7 risk factors modifiable through lifestyle changes that can help achieve ideal cardiovascular health [55], has also been proposed as a potential tool for dementia risk reduction. The LS7 risk score includes four behavioral (smoking, diet, physical activity, body mass index) and three biological (fasting glucose, cholesterol, and blood pressure) factors. A lower LS7 score indicating poorer CVD health has been associated with a higher risk of dementia in a long-term (25 years) observational study, while adherence to the LS7 ideal cardiovascular health recommendations in midlife has been linked to lower dementia risk [56]. Another CVD risk score including age, systolic blood pressure, total cholesterol, high-density lipoprotein, smoking, body mass index, and diabetes has been suggested as a useful tool for identifying individuals at risk for cognitive decline and dementia [57].

The global vascular risk score (GVRS) was developed to test whether the addition of behavioral and anthropometric risk factors to traditional vascular risk factors can improve the prediction of clinical vascular events (e.g., stroke and myocardial infarction). The score combines age, sex, ethnicity, waist, alcohol consumption, smoking, physical activity, blood pressure, antihypertensive medication, peripheral vascular disease, blood glucose, and cholesterol. The GVRS has been associated with cognition, e.g., decline in global cognition, episodic memory, and processing speed over time, although this association seemed to be more pronounced in APOE ε4 non-carriers [58]. The GVRS has been suggested as a feasible tool for use in primary care settings [59].

All the abovementioned studies have been observational. So far, only one study has investigated the CVD risk scores in the context of clinical trials for dementia prevention, reporting that multidomain interventions designed for dementia risk reduction significantly improved CVD risk scores such as FINRISK and SCORE [41].

Although CVD risk scores seem promising as potential tools for dementia risk reduction, their testing and validation for this purpose are still far from the standards available in the field of cardiovascular prevention. An important issue is the longer- vs. shorter-term prediction of dementia risk. Studies on dementia risk scores have clearly shown that risk profiles in midlife can be very different from risk profiles at older ages, and especially in older individuals who are already closer to dementia onset [60]. The time between the onset of brain pathology and the onset of clinical symptoms is also the time when “silent disease” can affect a variety of vascular, metabolic, and lifestyle factors, i.e., reverse causality. This is the most likely reason why shorter-term observational studies (< 5 years) in older populations often report associations between factors such as low blood pressure, low BMI, or low cholesterol and increased likelihood of dementia [60, 61]. Such findings likely indicate markers on an ongoing dementia-related disease and not actual risk factors. It is currently unclear if and to what extent CVD risk scores can be applied in older populations. Their associations with different types of brain pathology are also not yet determined.

## Discussion

Dementia prevention is still relatively new compared with, e.g., cardiovascular prevention, and much work is still left to be done to reach the standards of evidence and level of organization for pragmatic CVD risk reduction programs. Emerging evidence from recent multidomain prevention trials indicates that optimal preventive effects may be obtained through a precision risk reduction approach, i.e., targeting an individual’s overall risk profile instead of separate risk factors, and tailoring the right interventions to the right people at the right time. Randomized controlled trials testing early dementia risk reduction interventions have an inherent design complexity that CVD trials do not have to deal with, particularly in terms of outcome definitions. While CVD outcomes targeted by preventive interventions tend to be acute, clearly identifiable events, this is not the case for outcomes related to dementia diseases that are chronic, slowly progressive, often insidious, and requiring more specialized assessments to detect (e.g., neuroimaging, CSF). In addition, it is not fully clear how much intervention exposure and in what format would be necessary for achieving optimal effects, or at least what minimal level of exposure would be needed for some benefit to still be derived from dementia risk reduction interventions. Moreover, since most of the multidomain interventions were conducted in high-income countries, it is not clear whether their results can be generalized to low- and middle-income countries and is therefore necessary to collect further evidence from different settings. Thus, longer-term randomized controlled trials are much needed to address these issues. One such example is World Wide-FINGERS (WW-FINGERS, currently about 35 member countries), the first global network for multimodal dementia prevention trials, where the FINGER intervention model is currently being tested, adapted, and optimized in different populations, and geographic and economic settings, and focus is also on data harmonization and joint planning of these worldwide trials [62]. A limitation of this review is that the literature search was conducted in the English language only, and other potentially relevant studies may have been missed.

An important point regarding the development and testing of dementia and/or CVD risk scores in the context of dementia risk reduction concerns how findings are reported in the literature. Standardized and transparent reporting is crucial to facilitate decision-making about the choice of the most suitable risk estimation tools for specific purposes. The TRIPOD statement (transparent reporting of a multivariable prediction model for individual prognosis or diagnosis) [63] was published in 2015, and these guidelines would need to be followed similarly to for example CONSORT guidelines for reporting clinical trials, or STROBE guidelines for reporting cohort studies.

### From research to implementation

Most risk reduction interventions have been conducted in a research setting. BHSs will allow to implement the risk reduction interventions in the real world by offering the opportunity for cognitively unimpaired users to actively act and reduce their chances of developing dementia in the future. Before implementing the risk reduction interventions, an accurate dementia risk profiling (assessing the genetic, lifestyle, and biological risk factors; [7]) is needed to tailor the interventions to individual BHS users.

The 2019 WHO guidelines for risk reduction of cognitive decline and dementia [3] have emphasized that the implementation of interventions for cardiovascular and lifestyle risk factors may be combined with existing for example CVD or diabetes prevention programs and targeted to relevant populations. For this purpose, it is crucial that healthcare staff are fully aware of the importance of prevention in general and dementia prevention in particular. A recent survey highlighted that about 62% of the healthcare professionals did not consider dementia as a disorder but a condition of normal aging [64]. For effective implementation of prevention programs, a resource-efficient way may be to combine dementia prevention with cardiovascular prevention which is substantially more advanced in knowledge, research, and implementation compared to the more recent field of dementia prevention. Also, shared risk factors between the two diseases can help the use of existing knowledge and services to advance the idea of dementia prevention from research to practice.

**Fig. 1:**
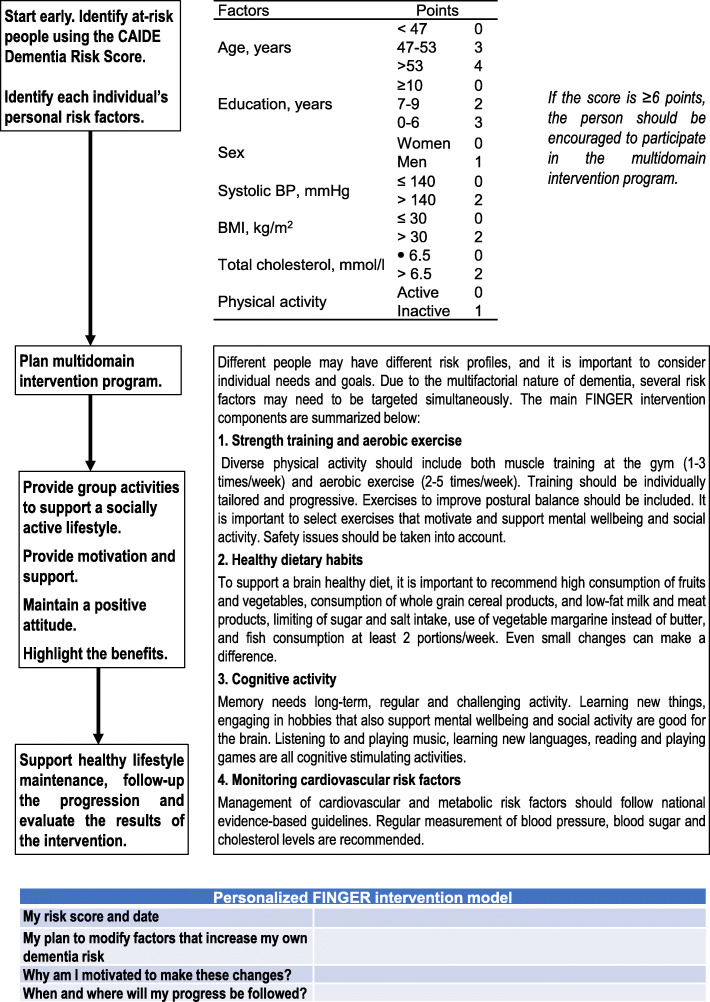
FINGER operational model for dementia risk reduction. The model was first published in Finnish by the Finnish Institute for Health and Welfare (http://urn.fi/URN:NBN:fi-fe2018092136291)

Engaging participants actively and in a meaningful manner is important in implementing prevention interventions. Large, longer-term multidomain intervention trials for dementia risk reduction have already shown that such interventions are feasible [16, 18, 19]. The first template for an operational model for dementia risk reduction has also been developed following the FINGER trial (Fig. 1). Although several factors such as higher age, poorer cognition, depressive symptoms, and smoking have been reported to be associated with lower adherence to multidomain interventions, results vary across the trials and different intervention components [65, 66]. Individually tailored approaches to risk reduction may also be more likely to ensure adherence. For example, a person at-risk may be compliant to a healthy diet but may need support with physical and cognitive activities, or another person with diabetes may need extra support for diet and management of other cardiovascular risk factors.

Initiating and maintaining healthy lifestyle changes in general are challenging at a personal level and is impacted by factors such as participants’ knowledge, access to facilities, time management, preference, and attitude towards prevention. Another layer of complexity is added especially when considering the implementation of such interventions or programs in low- and middle-income countries where prevention at mid-life may not be deemed as important as perceived in the Western world. Rosenberg et al. [67] recently studied the reasons for participation in a European multinational, multidomain eHealth lifestyle prevention trial (HATICE) targeting at-risk older adults without significant cognitive impairment. The participants were asked to specify the reasons for participation in the trial to which most responded: the desire to contribute to scientific progress, the possibility to improve their own health through lifestyle changes, and access to additional medical monitoring in the trial. Whether these same reasons motivate persons from other cultures and countries to participate and adhere to lifestyle interventions remains to be ascertained.

Therefore, it is important to identify the motivating factors, participants’ expectation, and extending support to them or their active participation. Some motivating factors for participants to join and engage in prevention programs could be personal goal setting for the maintenance of participants’ current and future health and avoidance of disability or dependency later in life [67]. Knowing their expectation during and after the participation would help educate them and gauge their goals and expectations realistically and for this those who are, e.g., at higher risk or lagging in motivation, to offer them extra support.

## Conclusion

Evidence on the efficacy of risk reduction interventions is promising, but not yet conclusive. More long-term multidomain randomized controlled trials are needed to fill the current evidence gaps, and the WW-FINGERS points in this direction. Nevertheless, consistent evidence suggests that a precision risk reduction approach may be most effective for dementia prevention. Such an approach can be implemented in BHSs.

## Data Availability

Data sharing is not applicable to this article as no datasets were generated or analyzed during the current study.

## Abbreviations

WHO: World Health Organization
CVD: Cardiovascular conditions
BHS: Brain Health Services
